# Light-activated shape morphing and light-tracking materials using biopolymer-based programmable photonic nanostructures

**DOI:** 10.1038/s41467-021-21764-6

**Published:** 2021-03-12

**Authors:** Yu Wang, Meng Li, Jan-Kai Chang, Daniele Aurelio, Wenyi Li, Beom Joon Kim, Jae Hwan Kim, Marco Liscidini, John A. Rogers, Fiorenzo G. Omenetto

**Affiliations:** 1 Silklab, Suite 4875, 200 Boston Avenue, Tufts University, Medford, MA USA; 2 Department of Biomedical Engineering, Tufts University, Medford, MA USA; 3 Center for Bio-Integrated Electronics, Northwestern University, Evanston, IL USA; 4 Dipartimento di Fisica, Università degli Studi di Pavia, Pavia, Italy; 5 Departments of Materials Science and Engineering, Biomedical Engineering, Neurological Surgery, Chemistry, Mechanical Engineering, Electrical Engineering and Computer Science, and Simpson Querrey Institute for BioNanotechnology, Northwestern University, Evanston, IL USA; 6 Department of Physics, Tufts University, Medford, MA USA; 7 Department of Electrical Engineering, Tufts University, Medford, MA USA; 8 Laboratory for Living Devices, Tufts University, Medford, MA USA

**Keywords:** Bioinspired materials, Photonic crystals

## Abstract

Natural systems display sophisticated control of light-matter interactions at multiple length scales for light harvesting, manipulation, and management, through elaborate photonic architectures and responsive material formats. Here, we combine programmable photonic function with elastomeric material composites to generate optomechanical actuators that display controllable and tunable actuation as well as complex deformation in response to simple light illumination. The ability to topographically control photonic bandgaps allows programmable actuation of the elastomeric substrate in response to illumination. Complex three-dimensional configurations, programmable motion patterns, and phototropic movement where the material moves in response to the motion of a light source are presented. A “photonic sunflower” demonstrator device consisting of a light-tracking solar cell is also illustrated to demonstrate the utility of the material composite. The strategy presented here provides new opportunities for the future development of intelligent optomechanical systems that move with light on demand.

## Abstract (web summary)

Programmable optical actuation in a material provides special possibilities for applications. Here, the authors combine photonic crystals with elastomers to provide material composites with tunable deformation and actuation as a function of moving light.

## Introduction

Light−matter interactions are sophisticatedly controlled by natural systems for efficient light harvesting, manipulation, and management utilizing micro-and nano-structural constructs built from a limited choice of constituent elements^1,2^. Functional outcomes include structural coloration/camouflage, vision, signaling, communication, thermal regulation, and photosynthesis, through effective manipulation of light reflection, diffusion, diffraction, transmission, and absorption^3–5^. These architectures have long been a source of inspiration for the development of multiple artificial optical functional materials for effective light-energy conversion^2,3,6^. Specifically interesting among these are periodic nanostructures (i.e., photonic crystals – PhCs) that can control and manipulate the flow of light by strongly suppressing the propagation of photons of specific frequencies through the structure^7,8^. These geometries have been successfully utilized as light-harvesting layers in various optical devices to enhance light-energy conversion, including photocatalysis^9,10^, photovoltaics^9,11^, light-emitting diodes^12,13^, and photothermal systems^14,15^.

Another light–matter interaction of interest converts optical energy into mechanical action, with several outcomes that add utility to applications in soft robotics^16,17^, biomedical devices^18,19^, or sensing^20^ because of the ability to remotely activate devices^21^. There has been exciting progress in the development of high performance optomechanical systems based on liquid crystal networks^22–24^, hydrogels^25,26^, shape memory polymers^27,28^, inequivalent expansion of gradient materials^29,30^, inorganic materials^31,32^, and others^33^. Current strategies for materials for optomechanical control involve the optimization of molecular structure assembly with outcomes that cover complex movement such as folding^23,30^, swimming^25,34,35^, walking^31^, self-oscillation^24,25^, and heliotropic motion^26,36^. Great efforts have been devoted in finding various approaches based on photothermal and photochemical mechanisms to manage the light-energy conversion that dominate the actuation^22,29,34,37^. Most optomechanical devices are engineered to perform local light-energy conversion^23,30,31^, which usually involve complex and energy-intensive fabrication process or complicated setups.

We describe here a composite material exploiting both modes of light–matter interaction in which the confluence of programmable photonic crystals with elastomeric materials enables a class of photo-responsive actuators that allow controllable and tunable actuation, and complex deformation in response to simple light illumination.

## Results

### Photonic optomechanical actuator

**Fig. 1:**
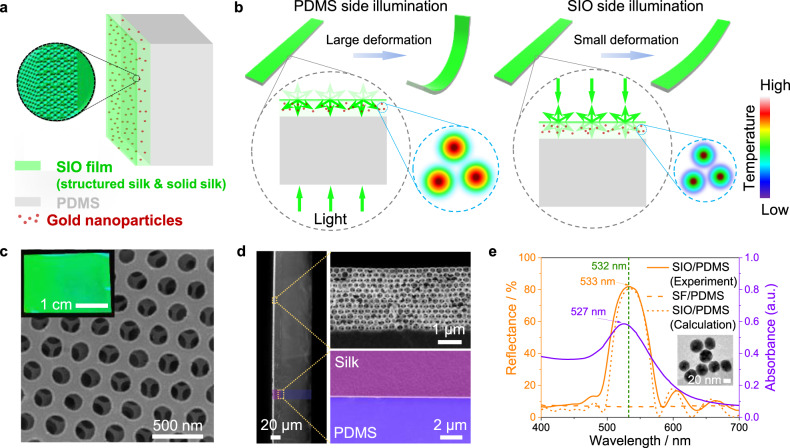
Structure, mechanism, and optical properties of photonic optomechanical actuator. **a** Schematic illustration of the structure of photo-deformable photonic bilayer film. The enlarged image shows the nanostructure of the inverse opal. **b** Schematics showing the mechanism of photonic-structure-controlled actuation. The photonic structure enhances (PDMS side illumination) or weakens (SIO side illumination) the gold-nanoparticles-driven photothermal conversion by controlling light propagation within the system. **c** Surface SEM image of SIO obtained from the colloidal crystals composed of polystyrene spheres with a diameter of 300 nm. The inset is a photograph of the as-prepared photonic bilayer film, showing bright green iridescence. The image was collected in the direction perpendicular to the SIO film. **d** Cross-sectional SEM images of the photonic bilayer film. The enlarged images clearly show the photonic crystal structure displayed by ordered, hollow silk fibroin structure with air holes on the wall (top) and the compactly contacted interface between silk and PDMS (bottom). **e** Measured and simulated reflectance spectra of the photonic bilayer film and absorbance spectrum of gold nanoparticles. The experimental curve is in agreement with the theoretical calculation. Inset shows a transmission electron microscopy image of the gold nanoparticles. Data for an unstructured SF/PDMS bilayer film is also shown for comparison.

The photonic actuator is a bimorph structure consisting of a silk inverse opal (SIO), doped with gold nanoparticles and polydimethylsiloxane (PDMS), as illustrated in Fig. 1a. Silk fibroin is chosen as the passive layer because of its versatility, flexibility, ease of functionalization, remarkable optical properties, nanoscale processability, and polymorphic features that allow for photonic crystal programmability^38,39^. Silk also possesses a negative coefficient of thermal expansion (CTE)^40^ which is suitable to interface with PDMS as the active layer because of its large coefficient of thermal expansion, and excellent durability against repeated deformation and high temperature^41^, on top of its optical transparency. The significant CTE difference between silk fibroin^40^ and PDMS^41^ allows for the generation of bending motion in response to temperature increases caused by the photothermal effect when the bimorph is exposed to light.

The formation of programmable SIO films is based on colloidal assembly of polystyrene nanosphere multilayers as templates as previously described^38,42,43^. Silk solution doped with gold nanoparticles is infiltrated in the template and allowed to solidify into a free-standing silk/polystyrene composite film. The resulting structure obtained after dissolving the polystyrene spheres in toluene consists of a free-standing gold-nanoparticle-doped SIO film with a nanostructured silk layer (enlarged image in Fig. 1a). The gold nanoparticles are mostly located in the solid part of the SIO film because of the dimensional constraints imposed by the nanostructure that are comparable to the dimensions of gold nanoparticles (>30 nm in diameter), which is crucial for the generation of direction-dependent deformation as described below. The bimorph structure is then prepared by casting the PDMS onto the flat side of the film via spin coating and subsequent drying.

The nanostructured silk layer provides a photonic crystal lattice layer which serves as an effective reflector, enhancing or weakening the interaction between gold nanoparticles and light as a function of its direction of incidence (Fig. 1b). This photonic layer only works when its stop-band matches the plasmonic absorption of gold nanoparticles. When illuminating the SIO/PDMS bilayer film from PDMS side, light with wavelength matching the stop-band is partially reflected back into the gold-nanoparticle-rich layer after reaching the thin nanostructured layer, leading to a longer light propagation path within the system and, thus, stronger interaction between gold nanoparticles and light. This causes larger deformation (Fig. 1b, left) when compared to films with totally mismatched stop-band or without photonic layer. By contrast, light incident from SIO side is partially reflected and does not penetrate the gold-nanoparticle-rich layer, resulting in weaker interaction between gold nanoparticles and light and, thus, less material deformation (Fig. 1b, right).

In order to maximize light–matter interaction the photonic crystal layer is designed to have a stop-band that is spectrally matched to the absorption peak of gold nanoparticles. Reflectivity of the photonic structure is increased by controlling its lattice constant and the number of nanosphere layers. The resulting structure is shown in Fig. 1c, d. A scanning electron microscopy (SEM) image of the surface of the SIO shows the highly ordered hexagonal arrays of air cavities with lattice constant (center-to-center distance of the air cavities, Λ) of 300 nm (Fig. 1c), while the cross-sectional SEM image of SIO shows twelve-layer highly ordered hollow silk fibroin spheres with air holes on the wall (Fig. 1d) and confers the SIO a bright green iridescent color. Analysis of the interface between silk and PDMS reveals uniform contact, thereby guaranteeing effective heat transfer and stable performance. The reflectance spectrum of SIO taken at normal incidence shows a narrow stop-band peak centered at *λ* = 533 nm (Fig. 1e), which is well matched to the absorbance peak of doped gold nanoparticles (*λ* = 527 nm). The reflectance intensity of the stop-band peak is shown to approach 80%, thus enabling higher discrimination between light propagating within the device and interacting with gold nanoparticles than when traversing a portion without an inverse opal structure and PDMS (SF/PDMS). This is confirmed by analyzing the photothermal conversion of the bilayers (Supplementary Fig. 1). As expected, illuminating the photonic bilayer film on the PDMS side and SIO side shows high and low temperature increase (and corresponding photothermal conversion efficiency), respectively, while the SF/PDMS sample shows intermediate values and no dependence on the illumination direction. Unless otherwise specified, all the SIOs considered here are twelve-layered inverse opals with Λ = 300 nm (i.e., green colored SIOs).

### Actuation performance of photonic bilayer

**Fig. 2:**
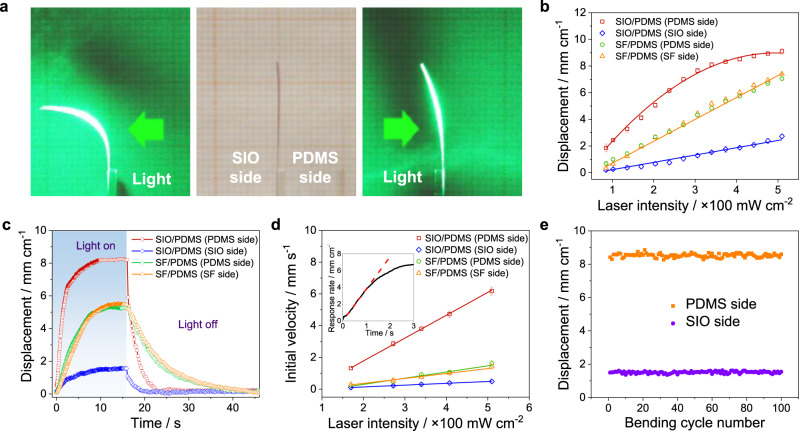
Actuation performance of the photonic bilayers. **a** Deformation of a photonic bilayer strip (20 × 2 mm) in response to a green laser (*I* = 17 m cm^−2^). **b** Dependence of maximal displacement on laser intensity. **c** Dependence of displacement on the laser illumination time (*I* = 35 m cm^−2^). **d** Response rate of bilayers in the initial deformation stage as a function of laser intensity. Inset shows the calculation of response rate, which is defined as the slope of the tangent line. **e** Dependence of the displacement on the bending cycle number of photonic bilayer film (*I* = 35 m cm^−2^). Error bars represent the standard deviation of the measurements (*n* = 5).

To evaluate light-induced deformations mediated by the photonic crystals on the bilayer films, small strips measuring 25 × 2 mm were fixed at one end and exposed to light. A green laser (*λ* = 53 nm) was used as the light source and directed perpendicularly to the sample surface (i.e., *θ* = 0°, Supplementary Fig. 2), unless otherwise noted. The strip illuminated on the PDMS side bends significantly more than when illuminated on the SIO side (Fig. 2a). Figure 2b plots the measured displacement at different illumination intensities. The displacement is defined as the straight distance at which the strip tip travels (Supplementary Fig. 3). Consistent with the results of heating (Supplementary Fig. 1a), PDMS side illumination on SIO/PDMS exhibits the largest displacement, while SIO side illumination shows the least. In comparison, SF/PDMS samples show intermediate deformation and the illumination direction does not affect the displacement. The evaluation of displacement induced by the laser illumination time was also examined (Fig. 2c). The displacement follows an exponential function when the laser is switched on and off. The response rate is evaluated from the initial slope of the displacement curve as a function of time after the light is turned on (Fig. 2d). SIO/PDMS samples with PDMS side illumination show the fastest response rate, while SIO side illumination gives the slowest response rate. The actuation of the photonic bilayer film is reversible with no observable deterioration in displacement after 100 cycles (Fig. 2e).

### Programmable shape-morphing

**Fig. 3:**
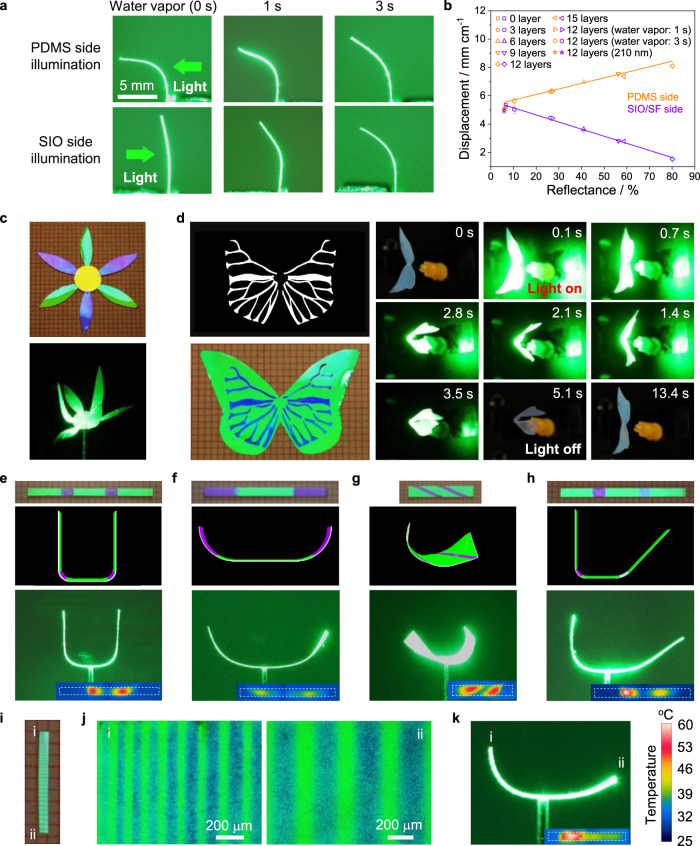
Programmable motion via photonic nanostructure design. **a** Photographs showing the deformation of the same photonic actuator before and after water vapor treatment for different durations (*I* = 35 m cm^−2^). **b** Dependence of displacement on the reflectance intensity of the SIOs at 532 nm (*I* = 35 m cm^−2^). **c** Photographs of a flower-shaped geometry (top) and relative selective bending of the photonic bilayer structure after light irradiation (bottom). **d** (Left) Stencil design used to produce a pattern on a butterfly-shaped photonic bilayer (top) and the corresponding photograph (bottom). The pattern was generated by exposing masked SIO to water vapor for 3 s. (Right) The actuation process of the butterfly under laser illumination from PDMS side (Input power: 0.5 W). **e**–**h** (Top) Photographs of patterned bilayer strips by water vapor exposure (**e**–**g** and **h**-left: 3 s, **h**-right: 1 s). The width of patterns is: 2 mm in **e** and **h**, 5 mm in **f**, and 1 mm in **g**. (Middle and bottom) Schematics and images of the corresponding motion modes under laser illumination from SIO side: symmetrical folding (**e**), outer bending (**f**), twisting (**g**), and unsymmetrical folding (**h**). **i** Photograph of a bilayer strip with microscale lines. The line width changes gradually from one side to another. **j** Optical microscopy images showing the lines near the two margin areas of the strip. **k** Corresponding motion mode upon exposure to laser source. The insets in **e**–**h** and **k** show the infrared images of the patterned strips under laser irradiation with dashed line outlining the edge of the sample before laser irradiation. Error bars denote the standard deviation of the measurements (*n* = 5).

While the ability to modulate actuation with the photonic structure is remarkable, the capability to design and control the geometric distribution of its photonic bandgap (position and intensity) provides further degrees of control over optomechanical actuation of the photonic bilayer film. This is first demonstrated by constructing SIOs with different nanostructures by either adjusting the number of layers and lattice constant of assembled colloidal crystals or by reconfiguring the photonic lattices after the formation of the inverse opal structure^38^ (Supplementary Fig. 4). For instance, Water-vapor-treatment induces the irreversible compression of photonic lattice in the weak vertical direction of green-colored SIO, leading to the modulation of the photonic stop-band and, thus, the decrease (PDMS side irradiation) or increase (SIO side irradiation) in the displacement (Fig. 3a). Such approach leverages the ability of water vapor to direct the polymorphic transitions of amorphous silk fibroin and provides the ability to design patterned photonic lattices with different responses^38,39^. Evaluation of the bilayer’s optical response shows that the displacement is linearly correlated with the reflected intensity at 532 nm (i.e., the wavelength of the laser source), independently of the SIO structure (Fig. 3b). The displacement of PDMS side illumination increases with the increase of reflectance, while it is just the reverse for SIO side illumination.

This photonic bandgap-dependent actuation enables the construction of complex 3D configurations by optically inducing controlled deformation of different 2D structures. As a first demonstration, a flower-like geometry was assembled using bilayers with different photonic lattices: a stamen made of a yellow SIO and six petals made of green and blue-violet SIOs (Fig. 3c). When the construct is illuminated from SIO side, the blue-violet petals bend dramatically towards the light source while the green petals only generate moderate bending (Supplementary Movie 1). Another example is a wing flap of a photonic butterfly was demonstrated by patterning the photonic crystals to generate green butterfly wings with blue veins through selectively exposing part of the shaped bilayer to water vapor using stencils (Fig. 3d). When the structure is illuminated the wings close rapidly and subsequently open gradually after the light is switched off (Supplementary Movie 2).

The capacity to programmably pattern the photonic crystal layers by inducing molecular rearrangement in the protein matrix enables the regulation of local light propagation within the bilayer system. This approach allows for local optomechanical actuation to realize different motion modalities. To demonstrate this idea, the photonic lattices in the bilayers were patterned as shown in Fig. 3e–h. When SIO sides are illuminated by the laser beam, symmetric folding (Fig. 3e), outer bending (Fig. 3f), twisting (Fig. 3g), and asymmetric folding (Fig. 3h) are generated since only the patterned areas are significantly deformed because of the enhanced interaction between light and gold nanoparticles (see also Supplementary Movie 3). This is further verified by thermal imaging to observe temperature distribution during actuation. From the IR images, the patterned areas reach higher temperatures than the unpatterned areas. In addition to these macroscale patterning examples, it is possible to obtain finer patterning details and manipulate light on the microscale. As a demonstration, a series of microscale lines gradually changing from 50 to 200 μm was designed on the photonic crystal, as shown in Fig. 3i, j. When this structure is illuminated, asymmetric bending is generated because of the heating gradient in response to the pattern (Fig. 3k). It is worth noting that water-vapor-based non-contact patterning method presented here allows for the creation of arbitrary, high-resolution (few microns) patterns, of which the dimension should be determined according to the geometry of the photonic bilayer to achieve desired deformation.

### Phototropic moving

**Fig. 4:**
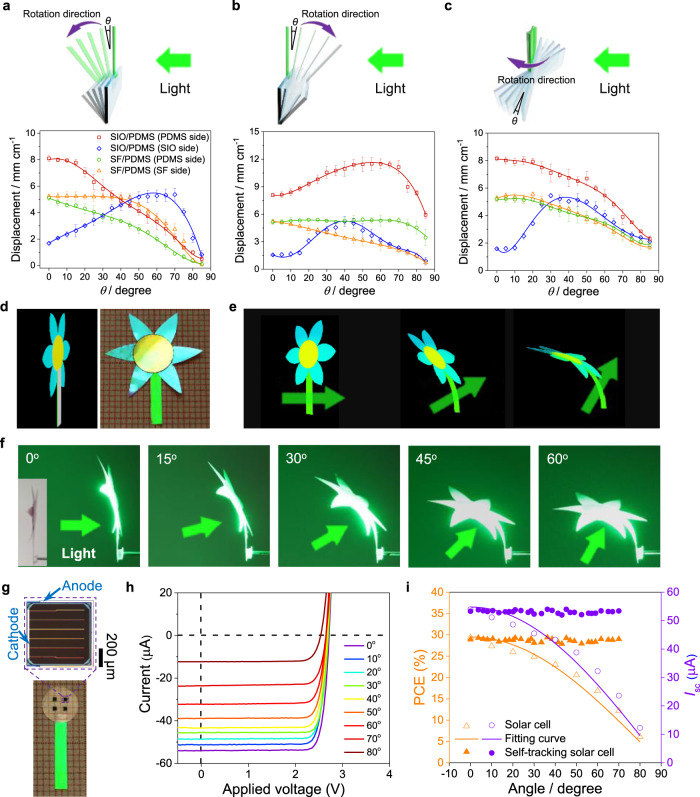
Angle-dependent actuation and phototropic moving. **a**–**c** (Top) Schematics of the rotation direction of the sample with respect to the laser source. The sample was illuminated from either PDMS or SIO/SF side. (Bottom) Dependence of displacement of the bilayers on the rotation angle (*θ*) of the sample (*I* = 35 m cm^−2^). Same legend for **a**, **b**, and **c**. **d** Schematic (left) and photograph (right) of a photonic sunflower. **e**, **f** Schematics (**e**) and images (**f**) showing different deformation states of the artificial sunflower with different illumination angles (*I* = 35 m cm^−2^). Insert in **f** indicates the initial state of the sunflower before illumination. **g** Photograph of the self-tracking photovoltaics system made of a photonic bilayer strip and an array of transfer-printed solar cells. Enlarged image shows the structure of one of the transfer-printed solar cells. **h** Current (*I*)–voltage (*V*) curves of the solar cell at different angles of incidence of light. **i** Measured power conversion efficiency (PCE) and short-circuit current (*I*_sc_) as a function of incident angle. Error bars indicate the standard deviation (*n* = 5).

While patterning photonic crystal layers effectively tailors the bandgap for controllable light manipulation, the angular dependence of the reflectivity (Supplementary Fig. 5a, b) allows for dynamic, reversible, and tunable optomechanical actuation. This is explored by evaluating the bilayer deformation at different illumination angles (Supplementary Fig. 2). Three actuation cases can be identified: (i) bilayer strip rotates perpendicularly to the short axis either away from (Fig. 4a) or (ii) towards (Fig. 4b) the light source, and (iii) the strip rotates perpendicularly to the long axis (Fig. 4c). The actuation of the photonic film with illumination angle is dramatically different from that of SF/PDMS film, especially for SIO side illumination whose displacement increases significantly at first and then decreases with the increase of illumination angle. Taking SIO side illumination of the photonic bilayer film in case (i) for instance, the displacement increases first until 70° because of the gradually enhanced light absorption and photothermal conversion (Supplementary Fig. 5c) with the blueshift of the stop-band and then decreases quickly due to the largely reduced amounts of light on the sample.

This feature is reminiscent of the heliotropic bending and moving of sunflowers with sunlight^44^ and could provide inspiration for tether free, light responsive materials that adapt and reconfigure themselves in response to moving light. As a demonstration, a photonic sunflower with pedicel composed of a photonic bilayer film but petals and stamen composed of SIO was designed (Fig. 4d). The schematics and images shown in Fig. 4e, f show the artificial sunflower continuously tracks the movement of a light source while its petals and stamen keep facing the light because of the continuous bending of its pedicel (see also Supplementary Movie 4). When the irradiation ceases, the sunflower returns to its initial position. In addition to bending tracking, phototropic twisting motion can also be achieved by locally attaching PDMS on SIO (Supplementary Fig. 6a). The lollipop-like geometry tracks the light source continuously with a twisting motion (Supplementary Fig. 6b, c, see also Supplementary Movie 5).

This wireless, light responsive, phototropic system has utility as a light tracking device and could be used to enhance light-to-energy conversion efficiency when interfaced to photosensitive systems. As a demonstration, an array of transfer-printed solar cells (weight: ~20 µg for each one) is integrated with the photonic bilayer film (Fig. 4g). The solar cell light conversion efficiency is angle-dependent (Fig. 4h and Supplementary Fig. 7), i.e., the power conversion efficiency (PCE) and short-circuit current (*I*_sc_) decrease gradually as the illumination angle is increased with respect to the photoactive surface (Fig. 4i). After integration with the phototropic photonic film, the solar cells track the light source continuously (Supplementary Movie 6) keeping the angle between the solar cells’ substrate and the laser beam nearly constant (Supplementary Fig. 8) and maximizing the efficiency of the solar cell as the light source moves. As shown in Fig. 4i, both PCE and *I*_sc_ of the self-tracking solar cells keep almost unchanged with varying the illumination angle. While this example is optimized for a specific wavelength, in a real-life scenario would have to be adapted to accommodate a flexible structural color filter^45^ or have a broad-band photonic structure^46^ in order to achieve heliotropic movement under sunlight illumination. In addition, the weight and size of the device that can be mounted in such photonic bilayer are determined by its geometry, which should be further optimized to improve its loading capacity for devices. Phototropic structures such as this could be also integrated with other solar energy utilization systems/devices, such as solar thermal collectors or artificial photosynthesis devices, to adaptively optimize their energy output.

### Reconfigurable actuation

Finally, given the confluence of reconfigurability of photonic structures and angle dependence of stop-band, the photonic actuators could provide interesting opportunities to alternate between different motion modes and realize switchable three-dimensional configurations by only tuning the illumination angle. The former is demonstrated by reexamining the motion modes of the patterned samples shown in Fig. 3e–h after being laterally exposed to laser beam (such as 45°). Interestingly, only overall bending is generated for all the samples because of the similar light capture within the patterned and unpatterned areas (Supplementary Fig. 9). The latter is demonstrated by reshaping the photonic bilayer into a cross-shaped geometry with two symmetric sides locally patterned using water vapor (Supplementary Fig. 10a). When the bilayer film is illuminated vertically from the SIO side (0°), only the patterned two sides fold (Supplementary Fig. 10b, Supplementary Movie 7), which is consistent with the anisotropic temperature distribution during actuation. However, if the sample is laterally exposed to laser beam (45°), the four sides fold simultaneously into a box-like shape because of distributed heating of both patterned and unpatterned regions (Supplementary Fig. 10b, Supplementary Movie 7).

## Discussion

The combination of a tunable biopolymer material (silk fibroin), an elastomer, and a reconfigurable photonic crystal structure provides complex modes of programmable optomechanical actuation by molding the flow of light into the composite structure. This strategy allows controlled spectral distribution at the microscale, offering the potential for photonic microactuators with complex deformation with potential utility for microrobotic technologies. The availability of a reconfigurable photonic crystal layer for effective, angle-dependent light harvesting opens up the possibility of generating optomechanical actuators that act both as back reflectors^11,47^ and solar trackers. The reconfigurability of the photonic crystal layer and the ease of functionalization of silk matrix can provide new avenues for optical devices with complex actuation by incorporating photothermal components with different absorption properties such as gold nanocrystals with different morphologies^48^ or carbon nanotube with selective chirality distributions^32^, and by selectively matching the wavelength of light source, photonic bandgap, and the absorption band. Considerable opportunities exist to expand this work to other optomechanical systems, like liquid-crystal elastomers, hydrogels, and shape memory polymers opening promising directions for future development of intelligent and multifunctional optomechanical devices.

## Methods

### Gold nanoparticles synthesis

Citrate stabilized gold nanoparticles were synthesized using the method described in literature^49^. Trisodium citrate dihydrate (S1804, Sigma-Aldrich, USA) and gold (III) chloride trihydrate (520918, Sigma-Aldrich, USA) were used without further modification. In a 50-mL beaker, 20 mL of 1.0 mM HAuCl_4_ aqueous solution was heated to boiling under stir. After the solution began to boil, 2 mL of 38.8 mM trisodium citrate aqueous solution was quickly added to the beaker. In about 10 min with continued boiling and stirring, the solution became deep red color. During the boiling, the volume of the solution decreased due to evaporation, so that DI water was added to keep the total solution volume near 22 mL. When the solution was a deep red color, the beaker was removed from the hotplate and allowed to cool to room temperature. The cooled solution was centrifuged at 17,500 rpm for 30 min or until supernatant is clear. The supernatant was discarded, and 10 mL DI water was added to the tube to make AuNPs stock solution. The prepared solution was sonicated for 20 min before use each time to suspend the nanoparticles fully.

### Gold-nanoparticles-doped silk solution preparation

The regenerated aqueous silk fibroin solution was prepared using the established protocols^50^. *Bombyx mori* silk cocoons were boiled in a 0.02 M Na_2_CO_3_ water solution for 30 min to remove the sericin. The remaining fibroin was then rinsed in deionized water and allowed to dry for 2 days. The dried fibroin was dissolved in 9.3 M LiBr solution at 60 °C for 4 h, followed by a dialysis process against deionized water for 3 days to obtain a 7–8 wt% silk fibroin solution in water. Gold-nanoparticles-doped silk solutions were prepared by mixing as-prepared silk solution with 150 µL gold nanoparticles solution. Gentle agitation was applied to obtain a uniform dispersion.

### Gold-nanoparticles-doped silk inverse opal (SIO) films preparation

The gold-nanoparticles-doped SIO films were prepared by using polystyrene colloidal crystal multilayers as templates. The detailed fabrication method was described in refs. ^38,39,46^ and reproduced here in summary. Briefly, 30 µL suspension (4%) of monodisperse polystyrene spheres (with diameter of 210 nm or 300 nm) was introduced to the water surface to form a floating monolayer. After removing the spheres immersed into the subphase of water, large-scale hexagonally close-packed polystyrene monolayer array was formed at the water/air interface with the aid of sodium dodecyl sulfate. This monolayer was then transferred from the water surface to the surface of a polymethyl methacrylate-precoated Si wafer. By repeating the transferring procedure, colloidal crystal multilayers with desired layer numbers were obtained. The silk solution doped with gold nanoparticles was carefully cast onto the colloidal crystal multilayers to fill all the air voids. The sample was left to dry for 12 h (at 25 °C, 30–40% relative humidity) to form a composite film. The gold-nanoparticles-doped SIO films with thickness of ~15 µm were finally obtained after removing the polystyrene templates in toluene. Gold-nanoparticles-doped silk flat films with thickness of ~15 µm were also prepared as control samples.

### Fabrication of bilayer films

The bilayer films were prepared by casting PDMS (base agent and cure agent are of 10:1 weight ratio) onto the SIO (non-nanostructured surface) or SF film via spin coating (1300 rpm × 30 min) and then allowed to cure at 60 °C for 12 h. The thickness of the PDMS is ~60 µm.

### Fabrication of patterned SIO/PDMS bilayer films

Water vapor treatment was used to design various patterns. Stencils with various designs and sizes were first positioned on the surface of the SIO/PDMS films from the SIO side. Then, the masked bilayer films were placed above a heated water surface (about 40 °C) to directly expose the SIO surface to water vapor for a set of different durations. The distance between the films and water surface was fixed at 5 mm.

### Fabrication of solar cells

With a sacrificial layer of AlInP to aid the epitaxial lift-off (ELO) processes, a lattice-matched tandem structure of InGaP/GaAs/InGaAsNSb was epitaxially grown on a GaAs substrate to build printable three-junction (3 J) solar μ-cells^51^. Photolithography and wet etching processes define an active area of 650 × 650 µm^2^ for the 3 J cells and create trenches among cells for ELO process. Then, metal deposition of Ge/Ni/Au layers formed metal grid/fingers for cathode and anode contacts in recessed geometries. A patterned photoresist (8 µm) across the trenches served as anchors and etching barrier during the ELO process, where the AlInP sacrificial layer was removed by hydrochloric acid to release the 3 J cells from the substrate. Microscale transfer printing utilizing a patterned elastomeric stamp enables the integration of 3 J cells onto PDMS substrate^52^. Acetone rinsing and plasma aching was finally utilized to remove the photoresist anchors/residues.

### Structural and properties characterization

FESEM (Zeiss Supra55VP) was used to observe the surface and cross-sectional morphology of the bilayer film. TEM (JEOL, 2100) was used to observe the morphology of gold nanoparticles. The reflectance spectra were recorded using a fiber-optic spectrometer (USB-2000, Ocean Optics). The absorption spectrum of gold nanoparticles was measured with a spectrophotometer (V-570, Jasco). Photographs and movies were taken using a DSLR camera (Canon EOS rebel T1i). Temperatures and IR thermal images were recorded using an infrared camera (SC645, FLIR^®^Systems, Inc., Sweden). The spatial maximum temperature across the whole sample was recorded and averaged over a one minute period after the temperature reading was stabilized. For each laser intensity, three samples were measured and the mean value of their temporally averaged temperatures was calculated. The thermal degradation experiment was performed with a thermogravimetric analyzer (Q500, TA Instruments, USA) at a heating rate of 20 K/min. Specific heat capacity was measured with a differential scanning calorimeter (Q100, TA Instruments, USA) at a heating rate of 20 K/min. For the performance characterization of solar cells, thin film metallization of Cu followed by photolithographic patterning was performed to establish the terminal interconnects and pads for probing. The printed 3 J cells were characterized by a Keithley 2400 source meter under AM1.5 G illumination with an irradiation intensity of 100 mW cm^−2^. A manual tilt stage facilitates the angular dependent analysis of cell performance, where the angle of incidence is determined by the calibrated tilt with respect to the solar simulator (Newport). The photovoltaic properties of self-tracking solar cell at different illumination angles were measured by leveraging the angle value between the solar cells’ substrate and the laser beam at corresponding illumination angle.

### Displacement measurement and data analysis

The tested samples had their edges taped onto a glass slide which was fixed to an optical mount. The illumination area of a collimated green laser with wavelength of 532 nm (Millennia Pro™, Spectra-Physics, USA) was expanded by a lens to cover the entire sample. The beam waist was measured by the knife-edge method. For actuator displacement analysis the beam waist radius was 4.32 mm. The total output power *P*_total_ of the laser was measured by an optical power meter (PM100A, Thorlabs GmbH, Germany). The received power (or input power, *P*_input_) on the sample surface from a Gaussian laser beam was calculated by:

1 $$P_{{\mathrm{input}}} = (1 - R){\int}_{ \!\!\!\!- w/2}^{w/2} {{\int}_{\!\!\!\!-l/2}^{l/2} {I_0\exp \left( { - \frac{{2(x^2 + y^2)}}{{r^2}}} \right)dxdy} }$$

where *R* is the reflectance of the sample surface, *r* is the beam radius, *I*_0_ is the intensity amplitude of the laser calculated by $I_0 = 2P_{{\mathrm{total}}}/\pi r^2$ for Gaussian beam profile, *w* and *l* are the sample width and length, respectively.

The illumination intensity (*I*) was calculated by:

2 $$I = P_{{\mathrm{input}}}/A$$

where *A* is the area of the sample.

The deformation of the bilayer during actuation was recorded by a DSLR camera (Canon EOS rebel T1i) at a frame rate of 30 fps and the tip movement was analyzed using Matlab^®^. The length of the samples (1 cm) was used to calibrate the pixel length in order to calculate the displacement. All the displacement measurements were performed at 20–25 °C and 20–30% RH.

### Optical simulation

The numerical simulation of the SIOs was carried out using rigorous coupled-wave analysis (RCWA)^38^. We considered a 12-layer SIO consisting of a silk matrix with spherical air holes lying on a substrate made of silk and gold nanoparticles. The presence of gold nanoparticles was taken into account by means of an effective dielectric function calculated using the Maxwell-Garnett model, in which the porosity corresponds to the concentration of the gold nanoparticles dispersed inside the silk matrix. The theoretical reflectance spectrum was then fitted to the experimental data by taking the lattice constant Λ as the only free parameter, leading to an estimated value Λ = 282 nm. Since in the experiment we used non-polarized light, the theoretical spectra are obtained by averaging TE and TM reflectance spectra, which have been calculated for angles of incidence in the range from 0° to 45° with an interval step of 5°.

### Calculation of photothermal conversion efficiency

The method used for calculating photothermal conversion efficiency is elaborated in refs. ^28,53,54^ which is briefly introduced here. The photothermal conversion efficiency is defined as the ratio of thermal energy *Q* to input light energy *q*:

3 $$\eta = \frac{Q}{q}$$

The generated heat *Q* raises the sample temperature to *T*_max_ from environmental temperature $T_{{\mathrm{room}}}^{{\mathrm{max}}}$ and can be expressed by:

4 $$Q = UA(T_{{\mathrm{max}}} - T_{{\mathrm{room}}}^{{\mathrm{max}}})$$

where *U* is heat transfer coefficient, *A* is the sample surface area, and $T_{{\mathrm{room}}}^{{\mathrm{max}}}$ refers to the maximum air temperature surrounding the sample. Rate constant of the energy loss is defined as:

5 $$w = \frac{{UA}}{{Cm}}$$

where *C* is mass-specific heat capacity and *m* is the mass of the sample.

By fitting the cooling curve of the experimental results with:

6 $$T\left( t \right) = \left( {T_{{\mathrm{max}}} - T_{{\mathrm{room}}} - a} \right)\exp \left( { - wt} \right) + a \cdot \exp \left( { - w_{{\mathrm{surr}}}t} \right) + T_{{\mathrm{room}}}$$

with coefficient $$a = \frac{w}{{w - w_{{\mathrm{surr}}}}}\left( {T_{{\mathrm{room}}}^{{\mathrm{max}}} - T_{{\mathrm{room}}}} \right)$$

where *w*_surr_ is the rate constant of the air surrounding the sample, and *T*_room_ is the air temperature surrounding the sample at *t* = 0 (i.e., room temperature), one can obtain the value for *w*, *w*_surr_, and $T_{{\mathrm{room}}}^{{\mathrm{max}}}$. With known values for *C* and *m*, one can calculate the values for *U* and *Q*, and further acquire the photothermal conversion efficiency *η*.

### Calculation of angle-dependent photovoltaic properties

The open voltage is dependent on the incident angle with the following relation^55^

7 $$V_{{\mathrm{oc}}(\theta )} = V_0 + C\ln \left( {{\mathrm{cos}}\theta } \right)$$

8 $$C = \frac{{mkT}}{e}$$

where *V*_0_ is the open circuit voltage at normal incidence, *m* is the non-ideal diode factor, *k* is the Boltzman’s constant, *T* is the temperature of the solar cell, and *e* is the electron charge. By fitting the *V*_oc_ data with different incident angles, we obtained the *V*_0_ value to be (2.698 ± 0.023) V and *C* is fitted to be (0.080 ± 0.03) V. The error bar is 95% confidence interval for all the fitting processes. The solar cell efficiency (*η*) also known as the power conversion efficiency (PCE) is defined as the ratio of power generated by the cell and the input light power. The generated power can be calculated with open circuit voltage *V*_oc_, short circuit current *I*_sc_, and fill factor *FF*.

9 $$\eta = \frac{{V_{{\mathrm{oc}}}I_{{\mathrm{sc}}}FF}}{{P_{{\mathrm{in}}}}}$$

*FF* is independent of incident angle, while *I*_sc_ obeys Lambert’s cosine law^56^, thus

10 $$I_{{\mathrm{sc}}}(\theta ) = I_0{\mathrm{cos}}\theta$$

where *I*_0_ is the short circuit current with normal light incidence. With both *V*_oc_ and *I*_sc_ being angle dependent, the efficiency can be written as

11 $$\eta \left( \theta \right) = \frac{{V_{{\mathrm{oc}}}\left( \theta \right)I_{{\mathrm{sc}}}(\theta )FF}}{{P_{{\mathrm{in}}}}} = \eta _0{\mathrm{cos}}\theta + \eta _0\frac{1}{{V_0}}C{\mathrm{ln}}({\mathrm{cos}}\theta ){\mathrm{cos}}\theta$$

By fitting the ***η***(***θ***) data, one obtains ***η***_0_ = (29.5 ± 1.74).

## Supplementary information

Supplementary Information

Description of Additional Supplementary Files

Supplementary Movie 1

Supplementary Movie 2

Supplementary Movie 3

Supplementary Movie 4

Supplementary Movie 5

Supplementary Movie 6

Supplementary Movie 7

## Data Availability

The data that support the findings of this study are available within the main text and the Supplementary Information. All data are also available from the corresponding authors upon reasonable request.
