# Supplementary material for: Light-activated shape morphing and light-tracking materials using biopolymer-based programmable photonic nanostructures
Source: Nat Commun. 2021 Mar 12;12:1651. doi: 10.1038/s41467-021-21764-6 (PMC7955034; doi:10.1038/s41467-021-21764-6)
Supplement: Supplementary file 1 — Supplementary Information [file 41467_2021_21764_MOESM1_ESM.pdf]

Supplementary Information

Light-activated shape morphing and light-tracking materials using biopolymer-based programmable photonic nanostructures

Yu Wang[1,2,9], Meng Li[1,2,9], Jan-Kai Chang[3], Daniele Aurelio[4], Wenyi Li[1,2], Beom Joon Kim[1,2], Jae Hwan Kim[3], Marco Liscidini[4], John A. Rogers[3,5] and Fiorenzo G. Omenetto[1,2,6,7,8]*

[1]Silklab, Suite 4875, 200 Boston Avenue, Tufts University, Medford, MA 02155, USA.

[2]Department of Biomedical Engineering, Tufts University, Medford, MA 02155, USA.

[3]Center for Bio-Integrated Electronics, Northwestern University, Evanston, IL 60208, USA.

[4]Dipartimento di Fisica, Università degli Studi di Pavia, via Bassi 6, 27100 Pavia, Italy.

[5]Departments of Materials Science and Engineering, Biomedical Engineering, Neurological Surgery, Chemistry, Mechanical Engineering, Electrical Engineering and Computer Science, and Simpson Querrey Institute for BioNanotechnology, Northwestern University, Evanston, IL 60208, USA.

[6]Department of Physics, Tufts University, Medford, MA 02155, USA.

[7]Department of Electrical Engineering, Tufts University, Medford, MA 02155, USA.

[8]Laboratory for Living Devices, Tufts University, Medford, MA 02155, USA.

[9]These authors contributed equally to this work: Yu Wang, Meng Li.

*Corresponding author. E-mail: Fiorenzo.Omenetto@tufts.edu

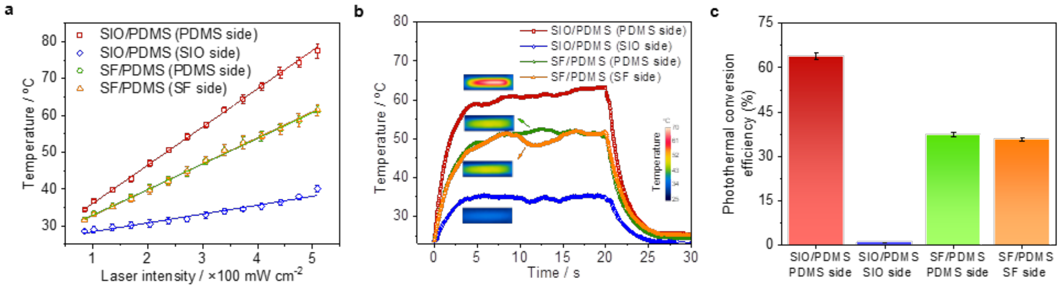

Supplementary Fig. 1. Photothermal conversion of bilayer films. a, Measured temperature increases as a function of laser irradiance. SIO/PDMS sample is the most and least heated when PDMS side and SIO side illumination are performed, respectively, while the SF/PDMS sample shows intermediate values and no dependence on the illumination direction. **b**, Measured temperature increases as a function of laser illumination time ($I = 350$ mW cm^{-2}). The temperature of SIO/PDMS sample with PDMS side illumination increases rapidly from room temperature (~25 °C) to a maximum temperature of ~63 °C, but it slowly rises to ~30 °C for SIO side illumination. The temperature of SF/PDMS film increases to ~51 °C at a moderate speed. Insets show the corresponding Infrared images after laser irradiation for 20 s. **c**, The calculated photothermal efficiency of the bilayer films. The photo-thermal conversion efficiency η reaches 63.83% ± 1.1% for SIO/PDMS film with PDPS side illumination, while it is only 0.80% ± 0.06% when SIO side is illuminated. The SF/PDMS film has similar and middle η values for both PDMS side (37.30% ± 0.7%) and SF side (35.64% ± 0.5%) illumination. Error bars denote the standard deviation of the measurements (n = 5).

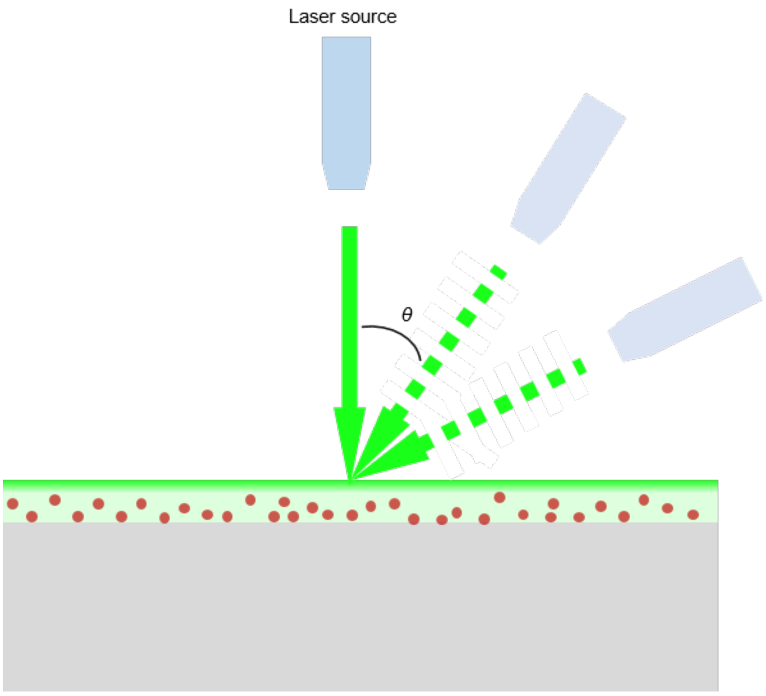

Supplementary Fig. 2. Schematic illustration of the bilayer sample illuminated at different angles. θ is defined as incident angle of laser beam.

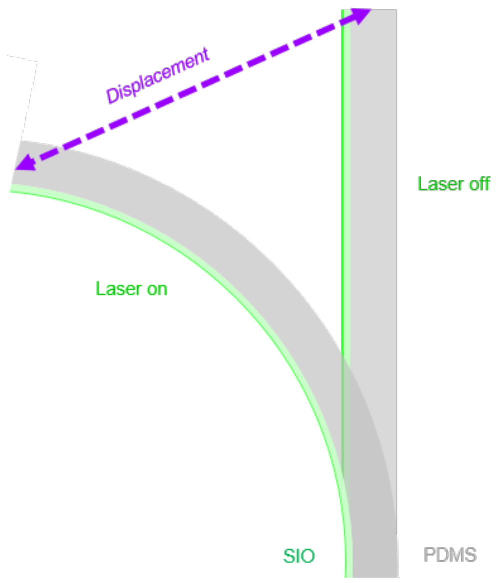

Supplementary Fig. 3. Schematic illustration of the calculation of displacement. The displacement is defined as the straight-line distance of the tip traveled.

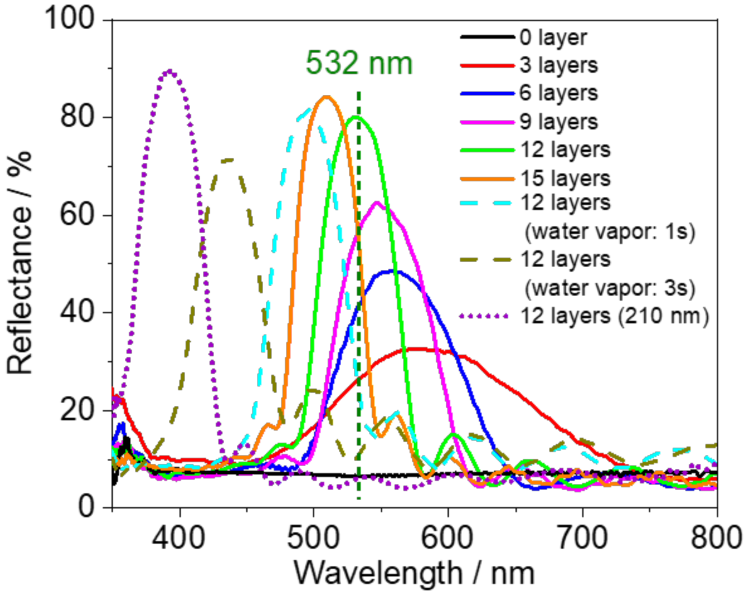

Supplementary Fig. 4. Reflectance spectra of photonic bilayer films with different photonic nanostructures. Different reflectance properties are generated by controlling layer numbers or lattice constants of SIOs or by reconfiguring the photonic lattices through contact-less water vapor exposure. The blueshift of the stop-band with the increase of the number of layers results from the shrinkage of the silk nanostructures in the out-of-plane vertical direction during immersion in toluene (to remove the template) and/or drying (to remove toluene). Such shrinkage occurred because silk in its amorphous configuration was used.

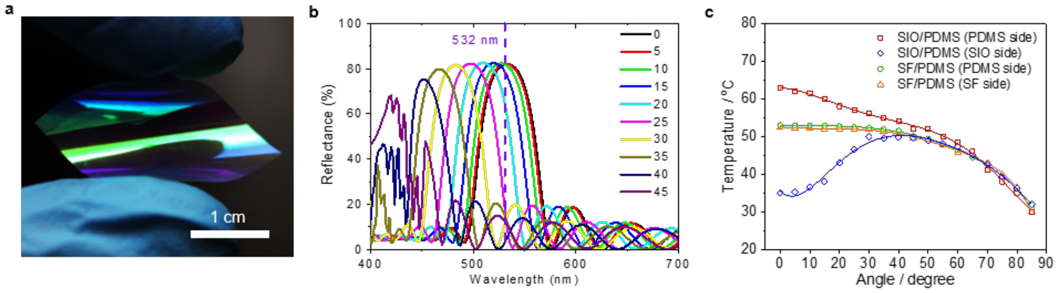

Supplementary Fig. 5. Angle-dependent reflection and heating. a, Photograph of a bent green colored photonic bilayer film showing different structural colors at different parts. **b**, Simulated reflectance spectra of green colored photonic bilayer film at different incident angles. The stop-band peak is gradually blue-shifted with the increase of incident angle, leading to the gradual decrease of reflection intensity at 532 nm (laser wavelength). **c**, Measured temperature as a function of laser illumination angle (I = 350 mW cm^{-2}). The temperature of the SIO/PDMS bilayer decreases gradually with angle for PDMS side illumination, while it increases first and then decreases if SIO side is illuminated. By comparison, the temperature of SF/PDMS bilayer decreases gradually regardless of illumination direction. Error bars denote the standard deviation of the measurements (n = 5).

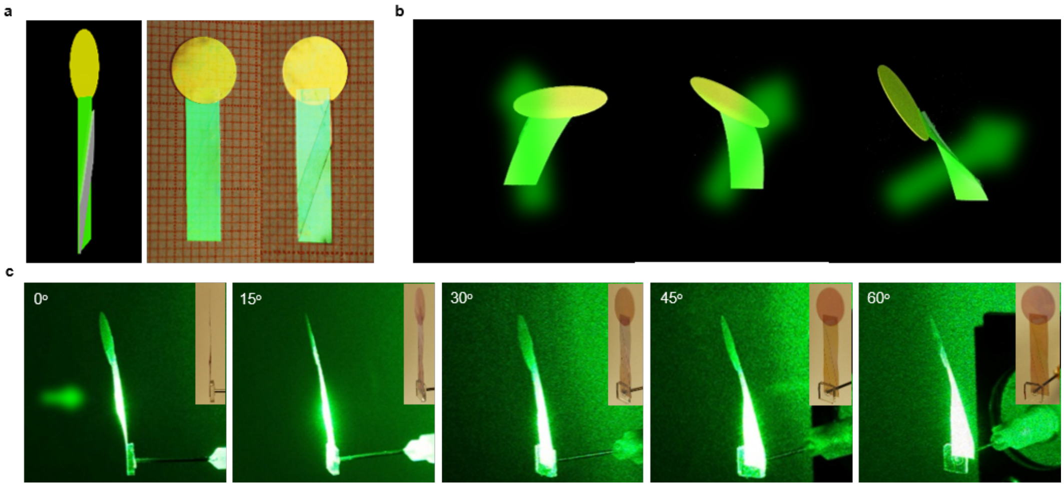

Supplementary Fig. 6. Phototropic twisting motion. a, Schematic and photograph of a lollipop-like geometry with the illustration of phototropic twisting movement. **b**, **c**, Schematics (**b**) and images (**c**) showing different deformation states of the lollipop-like geometry with different illumination angles. The sample tracks the light source continuously with the continuous twisting movement. Insets in (**c**) indicate the initial state of the sample at each angle before illumination.

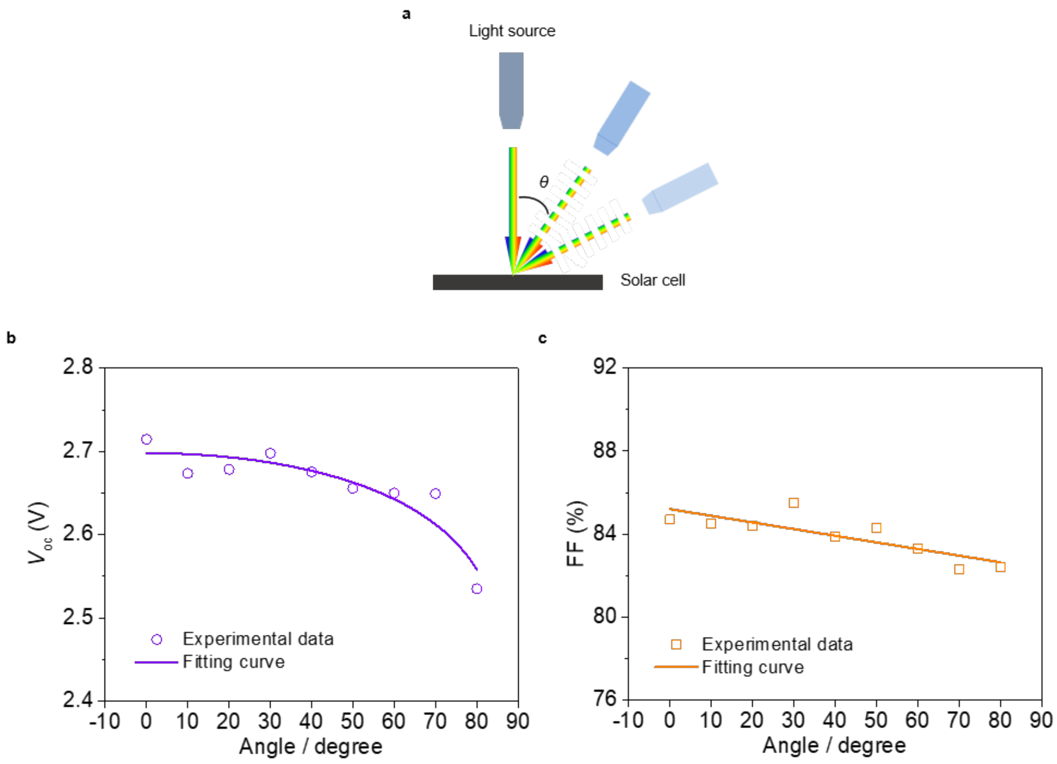

Supplementary Fig. 7. Performance of a microscale 3J solar μ-cell. a, Schematic showing the definition of illumination angle (θ). **b**, **c**, Measured open-circuit voltage V_{oc} (**b**), and fill factor FF (**c**) as a function of incident angle.

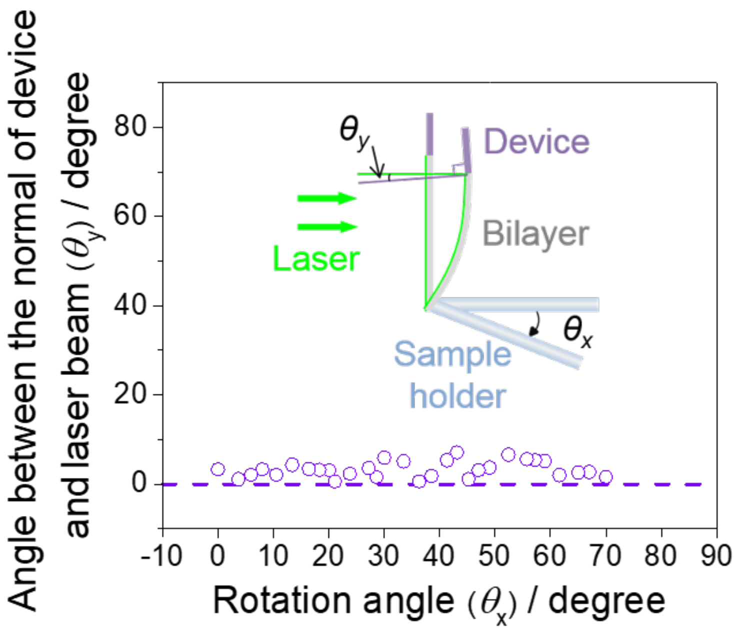

Supplementary Fig. 8. Angle between the normal of solar cells' substrate and laser beam (θ_y) as a function of rotation angle of the sample (θ_x). The angle between the device substrate and the laser beam almost keeps constant with the increase of rotation angle. The dashed line represents the angle of $\theta_y = 0^o$. Inset is the schematic of the definitions of θ_x and θ_y.

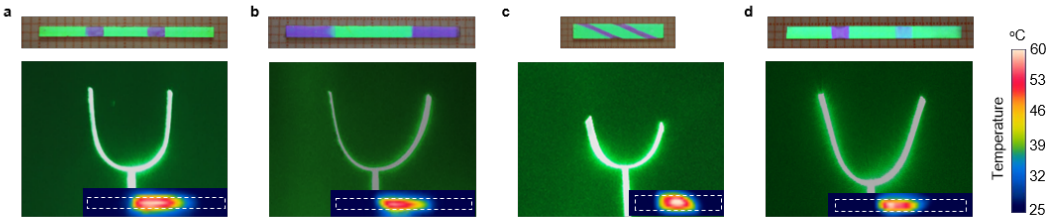

Supplementary Fig. 9 Photographs showing the pattern designs and the corresponding motion modes under laser illumination at 45°. a-d, Only bending is generated for all designs. Insets in (**a-d**) indicate the associated infrared images of the patterned strips under laser irradiation with dashed line outlining the edge of the sample before laser irradiation.

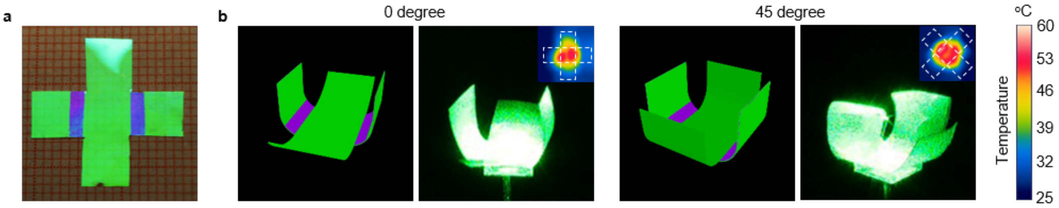

Supplementary Fig. 10. A self-folding box. a, Photograph of a cross-shaped piece of photonic bilayer film. **b**, Schematics and images showing the self-folding behavior under laser illumination at 0°and 45° from SIO side. Insets indicate the associated infrared images of the self-folding box under laser irradiation with dashed line outlining the edge of the sample before laser irradiation.

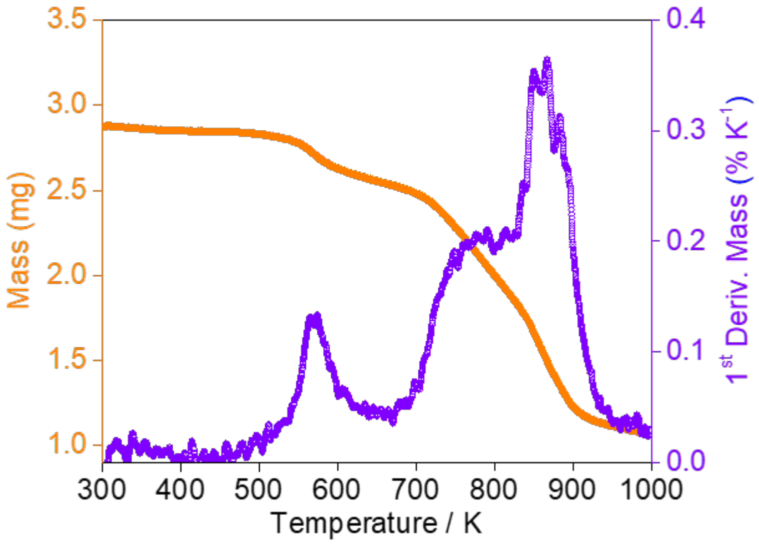

Supplementary Fig. 11. Thermal gravimetric results for photonic bilayer film. Water content in SF sample is ~0.70 wt% from mass loss below 373 K. The thermal degradation onset temperature is 573 K.

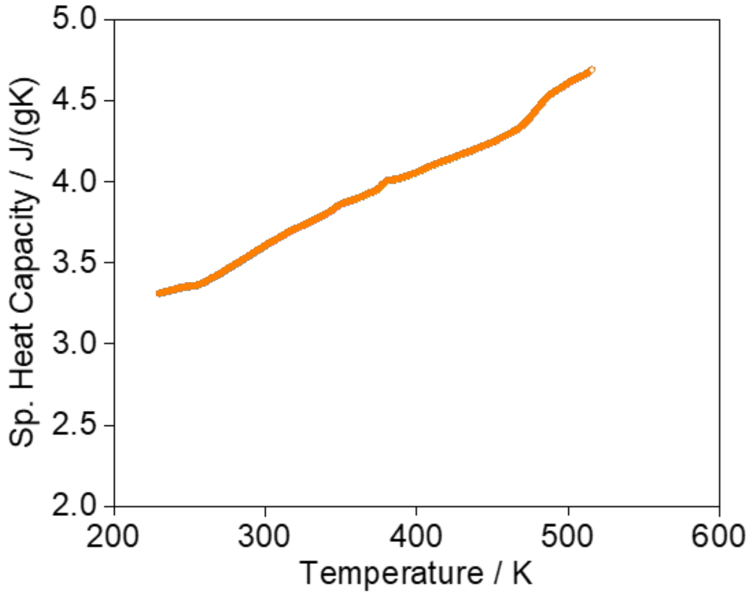

Supplementary Fig. 12. Specific heat capacity result for photonic bilayer film.
