# Supplementary material for: Light-activated shape morphing and light-tracking materials using biopolymer-based programmable photonic nanostructures
Source: Nat Commun. 2021 Mar 12;12:1651. doi: 10.1038/s41467-021-21764-6 (PMC7955034; doi:10.1038/s41467-021-21764-6)
Supplement: Supplementary file 2 — Description of Additional Supplementary Files [file 41467_2021_21764_MOESM2_ESM.pdf]

# Description of Additional Supplementary Files

File Name: Supplementary Movie 1
Description: Construction of complex 3D configurations - flower-shaped geometry. The blue-violet petals bend dramatically towards the light source while the green petals only generate slight bending.

File Name: Supplementary Movie 2
Description: Biomimetic locomotion - swing of butterfly wings. The wings close rapidly after light on and open gradually after light off.

File Name: Supplementary Movie 3
Description: Patterned bilayers for different motion modes: symmetric folding, outer bending, twisting, and asymmetric folding.

File Name: Supplementary Movie 4
Description: Phototropic bending movement of a photonic sunflower. The artificial sunflower tracks light source continuously with its petals and stamen keep facing the light.

File Name: Supplementary Movie 5
Description: Phototropic twisting movement of an artificial lollipop-like geometry. The lollipop-like geometry tracks the light source continuously with the continuous twisting movement.

File Name: Supplementary Movie 6
Description: Self-tracking photovoltaic system. The solar cells track light source continuously with the continuous bending movement of the photonic bilayer.

File Name: Supplementary Movie 7
Description: A self-folding box. 0° illumination causes the folding of the patterned two sides, while 45° illumination cause the four sides to fold simultaneously into a box-like shape.
